# Family-centred psycho-oncological counselling for parents with cancer: a longitudinal secondary data analysis of psychological distress, family functioning and parental concerns

**DOI:** 10.1186/s12885-026-17004-z

**Published:** 2026-09-22

**Authors:** Wiebke Geertz, Laura Inhestern, Isabelle Scholl, Corinna Bergelt

**Affiliations:** 1 Department of Medical Psychology, University Medical Centre Hamburg- Eppendorf (UKE), Hamburg, Germany; 2 German Centre for Child and Adolescent Health, partner site Hamburg, Hamburg, Germany; 3 Department of Medical Psychology, University Medicine Greifswald, Greifswald, Germany

**Keywords:** Adolescent, Cancer, Child, Counselling, Parent, Psycho-oncology

## Abstract

**Background:**

When a parent is diagnosed with cancer, it can place a significant burden on the entire family. The aim of this study is therefore to analyse the psychological distress experienced over time among cancer patients or their partners who are raising minor children and who utilize a family-oriented counselling service (COSIP), and to compare these data with those of a group receiving routine psycho-oncological care (PO).

**Methods:**

We conducted a secondary, longitudinal observational study based on routine care data from users of an outpatient psycho-oncological clinic. The database comprised assessments at baseline and at 6- and 12-month follow-ups, including measures of distress, anxiety and depression for COSIP and PO users. For COSIP users additional data on health-related quality of life (HRQoL), parental concerns, and family functioning was included. We conducted descriptive analyses and examined baseline group differences using t-tests and Pearson’s chi-square tests. Longitudinal trajectories were analysed using linear mixed-effects models.

**Results:**

Data from *n* = 34 parents who had registered themselves and their families for a COSIP counselling session between 2020 and 2022 were included in the analyses. Among the COSIP users, the time since the initial diagnosis was shorter on average (*p* = .017) and the parents were separated less frequently (*p* = .015) than in the group of PO users (*n* = 34). At baseline, PO and COSIP users exhibited elevated levels of psychological burden, with over 80% of parents reporting clinically significant distress, 54% presenting with moderate to severe anxiety symptoms, and 50% showing elevated levels of depressive symptoms. In the adjusted model, a significant main effect of time, for distress F(2, 107.24) = 14.72, *p* < .001, depression F(2, 69.61) = 12.27, *p* < .001 and anxiety F(2, 84.11) = 17.02, *p* < .001, but no group difference were found. We identified a significant effect of time for parental concerns F(2, 17.24) = 7.41, *p* = .006, parental HRQoL F(2, 31.52) = 4.34, *p* = .022 and children’s HRQoL F(2, 28.11) = 5.21, *p* = .012 among COSIP users.

**Conclusion:**

Parents who are confronted with a cancer diagnosis show high levels of psychological distress. Over time, a significant decrease in psychological distress among both groups and family-related outcomes in COSIP users was identified. Interestingly, no significant differences were observed between the groups, suggesting that the type of psycho-oncological support (individual or family-centred) may not differentially affect parental burden.

**Supplementary Information:**

The online version contains supplementary material available at https://doi.org/10.1186/s12885-026-17004-z.

## Background

About one in five people develop cancer in their lifetime [1]. Prevalence rates indicate that between 14 and 25% of cancer patients are parenting minor children. Thus, estimates reveal between 1.6 and 8.4% of children and adolescents have a parent with a history of cancer [2]. Families affected by cancer face a variety of new situations and challenges. The illness places a burden on all family members. However, children’s adaptation depends less on the cancer itself and more on the family’s coping processes and resources [3]. In families, uncertainties arise, for example, in the context of parent-child communication, especially concerning the illness [4, 5] and in communicating and dealing with individual needs [6]. Parents often notice signs of distress in their children, but sometimes avoid talking about their emotional reactions [7]. Previous research suggested that a cohesive, communicative and collaborative family system could have a protective effect on children’s mental well-being [8]. In addition, parents with cancer experience substantial parenting distress, including concerns about their children’s well-being, maintaining family roles, and coping with illness-related demands [9]. A systematic review also revealed significant psychological distress, with high rates of anxiety and depressive symptoms among cancer patients parenting minor children [10].

In order to meet the situation of affected families and to support them, specific counselling and care services exist nationally and internationally [11–14]. One of those interventions is the COSIP-concept (Children of somatically ill parents). It was developed in 2007 as a manualized, preventive, supportive intervention for families with a parent who is physically ill and was further specialized for the psycho-oncological setting in 2014 [3]. The programme aims to support children, parents, and the family system as a whole by addressing illness-related communication, parental concerns, family functioning, and children’s psychosocial needs. Further information on the COSIP concept can be found in Thastum et al. [15] and Romer et al. [3]. A setting for the whole family is particularly important as good communication and a supportive family environment that understands everyone’s needs appear to be important protective factors that facilitate the entire family’s adjustment to the illness [16].

Although family-focused interventions for families affected by parental cancer have been implemented for some time and are generally perceived as helpful, acceptable and feasible, a recent review indicates that only few interventions have undergone comprehensive evaluation, and evidence regarding the long-term sustainability of improvements in outcomes remains limited [9].

To address those research gaps the objective of this secondary analysis of longitudinal observational data was to assess the psychological distress among cancer patients or their partners who are raising minor children and registered for psycho-oncological support (individual or family-centred). Furthermore, this study aimed to examine family-related outcomes and their trajectories over time among COSIP users. For readability, the term “parents” is used throughout this manuscript to refer to both the parent with cancer and the healthy parent. The following research questions were addressed:

I. What is the level of psychological distress (including distress, depressive symptoms, and anxiety) among parents with minor children and a parental cancer diagnosis who register for COSIP?
II. How does psychological distress among COSIP users change over time, and how does it compare with users receiving routine psycho-oncological care (PO users)?
III. What are the levels of family-specific distress (including parental concerns, family functioning, parental health-related quality of life (HRQoL) and children’s HRQoL) among COSIP users, and how does the family-specific distress change over time?

## Methods

An exploratory secondary analysis was performed using longitudinal observational data derived from routine assessments conducted at baseline and at 6- and 12-month follow-up. The study employed a naturalistic design and included a sample of parents who enrolled in COSIP, alongside a matched comparison group of PO users at the specialized outpatient clinic. COSIP users were matched with PO users through a matching process based on age, gender, and role (patient versus healthy partner). This report was prepared in accordance with the STROBE reporting guidelines [17] (added as supplementary material). The study was approved by the local ethics committee (Local Psychological Ethics Committee at the Centre for Psychosocial Medicine, University Medical Centre Hamburg-Eppendorf; LPEK-0257).

### Measures

#### Demographic and disease-related variables

Demographic and disease-related data were obtained via self-report. Demographic variables comprised age, sex, number and age of children, and marital status. Disease-related variables included cancer diagnosis, presence of metastases, and time since diagnosis.

#### Psychological distress (assessed among COSIP and PO users)

##### Distress

The NCCN Distress thermometer is a screening instrument developed by the National Comprehensive Cancer Network (NCCN) to assess psychosocial distress in oncological patients [18]. It consists of a scale from 0 to 10, on which the experienced distress of the last week is reported. The internationally recommended cut-off of 5 or more was used as an indicator that a patient is in distress and needs support.

##### Depressive symptoms

Depressive symptoms were assessed using the PHQ-9. All 9 items can be rated on a 4‐point Likert‐scale from 0 to 3. The PHQ‐9 has good reliability and validity with higher sum scores indicating higher severity of depressive symptoms (total score range 0‐27). A total score of 10 or higher indicates possible depressive disorder [19].

##### Anxiety

Anxiety was assessed with the GAD-7, which uses a 4‐point Likert‐scale from 0 to 3. The GAD‐7 shows good psychometric properties. Total score range is 0–21 and higher sum scores indicate higher severity of anxiety symptoms. A total score of 10 to 14 indicates moderate anxiety symptoms, and a total score of 15 and higher indicates severe symptom levels [20].

#### Family-related distress (only assessed among COSIP users)

##### HRQoL

The HRQoL among COSIP users was measured using the German version of the global scale of the European Organization for Research and Treatment of Cancer Quality of Life Questionnaire (EORTC QLQ-C30). We used the *Global Quality of Life scale* as outcome, which is calculated by adding up the responses to two specific questions (question 29 and question 30) and transforming this sum linearly into a range from 0 to 100 with higher scores indicating better HRQoL. The EORTC QLQ-C30 is known to be reliable and valid and is widely used [21, 22].

##### Family functioning

Family functioning indicates the extent to which the family responds to the needs of its members [23]. The German version of the General Functioning subscale (GF12) of The McMaster Family Assessment Device (FAD) was used to assess family functioning. It consists of 12 items and it can be used alone as a global index and a valid general family measure. It is scored on a Likert scale from 1 to 4, higher scores represent more problems. The FAD shows good acceptance, reliability and validity [24].

##### Parenting concerns

The parenting concerns of cancer patients were measured with the German version of the Parenting Concerns Questionnaire (PCQ). The scale consists of 15 items that can be rated on a scale from 1 (not concerned) to 5 (extremely concerned). The PCQ proved a reliable and valid measure of parenting distress among cancer patients [25].

##### Children’s HRQoL

We assessed the children’s HRQoL using the proxy version of the KIDSCREEN-10 Index [26]. Parents answered the 10 items on a 5-point Likert scale (1 = not at all to 5 = extremely). We transformed the scores into T-values (M = 50, SD = 10, possible range 0-100) according to the scoring manual. Higher scores represent a higher health-related quality of life. Research has shown that the KIDSCREEN-10 is a reliable, valid, sensitive, and conceptually appropriate Quality of Life measure [27]. Although the original version of the KIDSCREEN-10 is intended for school children aged 6 to 18 years, we have also used it for younger children as other researchers have successfully done before [28].

### Interventions

COSIP and PO are part of the specialist care provided in an outpatient clinic at a comprehensive cancer centre in Germany.

#### COSIP – child-centred counselling for families

The conceptual framework for child-centred counselling for families affected by parental cancer was developed on the basis of the research conducted by the COSIP group [15]. The COSIP approach provides a flexible intervention framework that is tailored to each family’s individual needs, allowing both the number of sessions and the content to vary accordingly. The intervention is characterised by three phases: a diagnostic phase and an interventional phase, which comprises approx. 3–8 sessions, and transitions into a flexible concluding phase [3]. The manual provides specific guidance on conducting initial diagnostic interviews and formulating clearly defined intervention objectives. These objectives are tailored to the specific needs of each family. The focus of counselling can be on the family as a whole (e.g., promoting open communication), on the parents (e.g., strengthening perceived parenting skills) or on the child (e.g., strengthening the child’s active coping resources). COSIP counselling is provided by a child and adolescent psychotherapist or a psychotherapist who has completed training in the COSIP concept.

#### PO - Individual psycho-oncological treatment of the comparison group

The psycho-oncological outpatient clinic offers supportive psychotherapeutic care to cancer patients and their relatives, primarily delivered by clinical psychologists and certified psychotherapists trained in behavioural, humanistic therapy, or psychodynamic psychotherapy. Treatment mainly consists of one-on-one sessions with cancer patients or relatives, or may involve group sessions, couple sessions, or art and music therapy. Information on the specific treatments received by the patients included in this analysis was not available within the dataset.

### Participants

#### Inclusion and exclusion criteria

The participants comprised a sample of parents who were responsible for minor children and who had attended either at least one COSIP counselling session between January 2020 and December 2022 or at least one general PO appointment, and for whom data were available both at the time of the baseline survey and at 6-months follow-up. No restrictions were applied regarding cancer type, disease stage, family composition, or the nature of counselling concerns. As the questionnaire was part of a routine assessment and solely available in German, sufficient language skills were required for completion.

### Data collection and analysis

#### Procedure

The underlying data of this study were anonymized data based on a routine assessment conducted by the psycho-oncological outpatient clinic. Patients who wanted to attend COSIP counselling or PO were requested to complete and return a baseline questionnaire prior to their initial consultation. 6 months and 12 months following the initiation of counselling, patients were sent follow-up questionnaires to be returned in a stamped, self-addressed envelope. All patients provided their written consent prior to returning the routine assessment questionnaire, allowing their data to be further processed for research purposes.

#### Matching COSIP and PO group

Parents with minor children receiving PO were selected as a comparison group and descriptively matched to COSIP users based on age, gender, and role (patient or healthy partner). During the matching process, each parent in the COSIP group was matched with a parent from the PO group who corresponded in terms of these characteristics. Where possible, parents were matched directly by age; if an exact age match was not available, parents were matched within predefined 5-year age categories (e.g., 40–44 years). Despite the matching procedure, COSIP and PO users differed significantly at baseline regarding family status (*p* = .015, Cramer’s V = 0.35) and time since initial diagnosis (*p* = .017, d = 0.60) (Table 1). To account for these residual baseline differences, family status and time since diagnosis were included as covariates in the linear mixed-effects model (LMM). Therefore, between-group effects were estimated while statistically adjusting for these baseline characteristics (Table 1).

**Table 1:** Participant characteristics and comparison COSIP-users and PO-users at baseline

		COSIP users (*n* = 34)		PO users (*n* = 34)		Statistical comparison of COSIP and PO users	
		*N*	%	*N*	%	*P*-value	Cramers V/Cohen’s d
Role	Patient	27	79.4	27	79.4	matched	
	Relative	7	20.6	7	20.6		
Sex	Female	28	82.4	28	82.4	matched	
	Male	6	17.6	6	17.6		
Family status	single	4	11.8	7	20.6	0.015* ª	0.352
	married	30	88.2	21	61.8		
	divorced			6	17.6		
Highest level of education	Certificate of Secondary Education	3	8.8			.241ª	0.248
	General Certificate of Secondary Education	6	17.6	6	17.6		
	Vocational Diploma	4	11.8	2	5.9		
	General Qualification for University Entrance	21	61.8	26	76.5		
Cancer	Gastrointestinal	3	8.8	9	26.5	0.561 ª	0.358
	Breast	15	44.1	14	41.2		
	Brain	3	8.8	3	8.8		
	Lymphoma	4	11.8	2	5.9		
	Others (e.g., Lung, Brain, Gynecological)	9	26.5	6	17.6		
Cancer with metastases	no	25	73.5	23	67.6	0.728 ª	0.043
	yes	9	26.5	10	29.4		
	Information missing			1	2.9		

		Mean (SD)	Range	Mean (SD)	Range	*P*-value	Cramers V
Months since diagnosis		12.59 (21.08)	0–89	37.82 (56.19)	0-177	0.017* ᵇ	0.595
Number of sessions		5.33 (4.90)	1–18	n.a.	n.a.		
Age of the parent		44.59 (6.28)	34–56	44.94 (5.97)	32–57	matched	
Number of children		1.94 (0.65)	1–4	1.74 (0.71)	1–3	0.138 ª	0.285

ª pearson-chi-square -test

ᵇ t-test

*p < .05. **p < .01. ***p < .001

#### Data analysis

Missing data handling followed the scoring recommendations provided in the respective manuals for the EORTC QLQ-C30 and KIDSCREEN-10. For the PHQ-9, missing items were handled in accordance with previous research using the instrument, where missing values were replaced by the mean of the remaining items if fewer than 20% of items were missing [29]. This procedure was also applied to the PCQ, GAD-7 and FAD scales, given the low proportion of missing values [30]. The rationale for this approach was to maximize the available sample size while minimizing potential bias introduced by exclusion of participants through listwise deletion.

Descriptive analyses were performed to characterise the study sample and to identify the level of psychological distress (including distress, depressive symptoms, and anxiety) of COSIP users at time of registration (Research question 1) and follow-up assessments. To compare COSIP and PO users (Research question 2) trajectories of psychological distress (distress, anxiety, depression) across the three measurement time points were examined using LMM. The models included group, time, and the group-by-time interaction as fixed effects. Family status and time since first diagnosis were included as covariates to consider baseline differences between groups. Participant was modelled as a random intercept to account for the dependency of repeated observations within individuals. LMM were estimated using restricted maximum likelihood (REML). Repeated measurements were modelled with a first-order autoregressive [AR(1)] covariance structure. Family-related distress among COSIP users over time was analysed using LMM with a random intercept for participant to account for within-person correlations across three time points (Research question 3). The significance level was set at α = 0.05. Statistical analyses were performed using IBM SPSS Statistics Version 29.0.

## Results

### Sample characteristics COSIP user

**Fig. 1:**
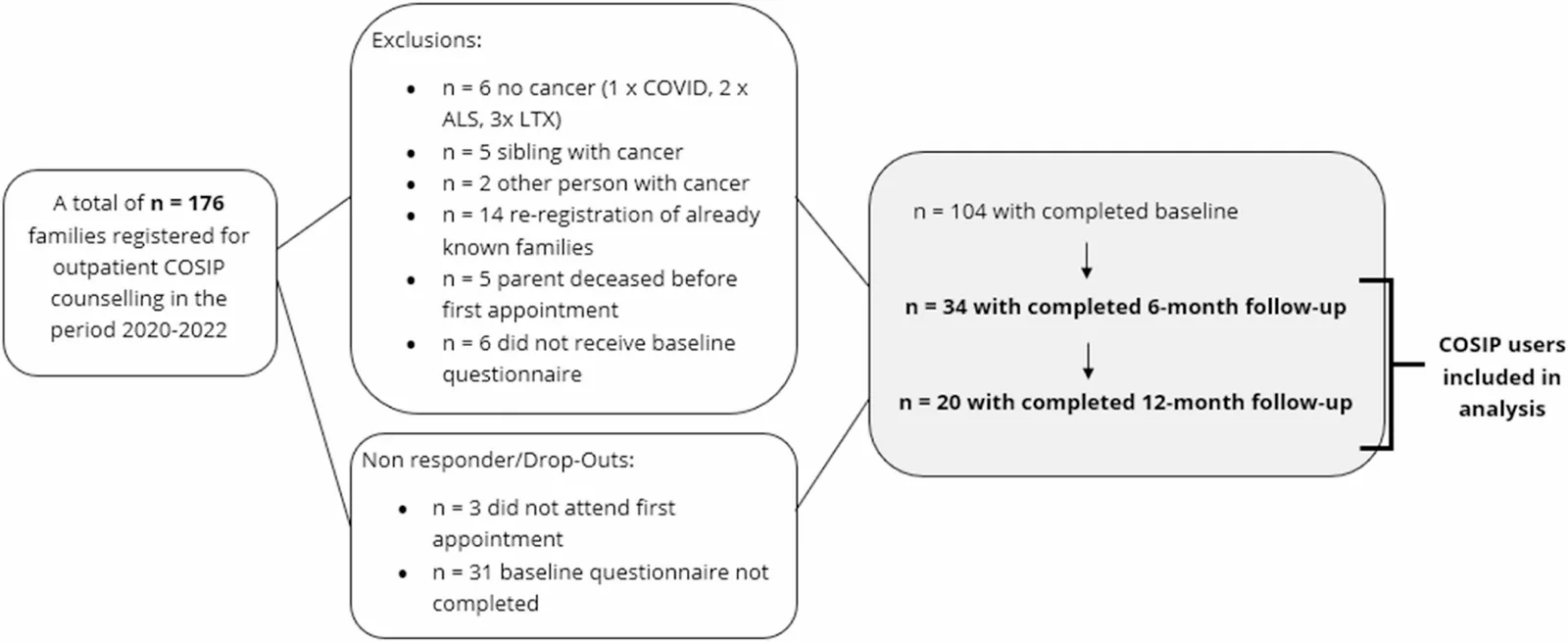
Flowchart overview of the inclusion process. Notes. ALS (Amyotrophic lateral sclerosis); LTX (Liver transplantation)

A total of 176 families registered for COSIP counselling between January 2020 and December 2022. 38 cases had to be excluded from the analyses due to the parent’s death prior to counselling, the parent having another life-threatening disease not cancer, or because instead of a parent, a sibling was diagnosed with cancer. 34 participants failed to respond after registration, while a further group did not complete the baseline questionnaire. Of the remaining 104 patients, 34 completed at least the first follow-up assessment and were consequently included in the analysis (Fig. 1). A high attrition rate was observed, with a substantial proportion of participants not completing the follow-up questionnaires, leading to a reduced sample size and a highly selected subgroup for the longitudinal analysis.

In the majority of included COSIP users (79%, *n* = 27), the ill parent registered for COSIP themselves. The participants were mostly female (82%, *n* = 28) and well educated, with over 60% of them having a General Qualification for university entrance (*n* = 21). The mean age of the parent who completed the questionnaire was 45 years (SD = 6.3, range 34–56). The mean number of children per family was 2 (range 1–4). The initial diagnosis occurred mostly within the last year (71%, *n* = 24) or the past five years (26%, *n* = 8). Breast cancer with 44% (*n* = 15), lymphoma with 12% (*n* = 4) and brain cancer with 9% (*n* = 3) were the most frequent cancer types. The mean number of counselling sessions was 5 (SD = 4.9, range 1–18) (Table 1).

A comparative analysis between follow-up completers and non-completers revealed no significant differences in baseline symptom severity of the psychological distress, or sociodemographic characteristics (i.e., gender and role) (see Supplementary Material, Appendix A).

### Descriptive analysis of psychological distress among COSIP users (Research question 1)

The level of general psychological stress among COSIP users was high at baseline and decreased over time. Based on established cut-off values, 82% of COSIP users met the criterion for elevated distress at baseline, with this proportion decreasing to 65% at 6 months follow up and 24% at 12 months follow up. Moderate to severe anxiety symptoms were reported by 50% at baseline, 32% at 6 months follow up, and 12% at 12 months follow up; corresponding values for depressive symptoms were 44%, 32%, and 9%, respectively (Table 2).

**Table 2:** Descriptive report of the psychological distress levels among COSIP users and PO users

Outcome		Baseline			After 6 months			After 12 months		
		*N*	M (SD)	Participants above cut-off *N* (%) ª	*N*	M (SD)	Participants above cut-off *N* (%) ª	*N*	M (SD)	Participants above cut-off *N* (%) ª
Distress	COSIP	34	7.21 (2.21)	28 (82.4)	32	6.03 (2.25)	22 (68.8)	14	4.93 (2.52)	8 (57.1)
	PO	34	7.29 (2.05)	31 (91.2)	34	5.53 (2.4)	23 (67.6)	17	6.24 (2.33)	13 (38.2)
Anxiety	COSIP	34	10.94 (5.94)	17 (50)	34	7.71 (4.59)	11 (32.4)	15	7.06 (4.71)	4 (11.8)
	PO	34	10.15 (5.08)	20 (58.8)	34	7.76 (3.93)	11 (32.4)	18	7.33 (5.83)	5 (14.7)
Depression	COSIP	34	9.26 (5.17)	15 (44.1)	34	8.03 (5.69)	11 (32.4)	15	6.87 (4.81)	3 (8.8)
	PO	34	10.94 (6.52)	19 (55.9)	34	8 (4.44)	11 (32.4)	18	6.05 (4.32)	5 (14.7)

ª Distress (DT score) cut-off ≥ 5, Anxiety (GAD-7 score) cut-off ≥ 10, Depression (PHQ-9 score) cut-off ≥ 10

### Psychological distress over time among COSIP users compared with PO users (Research question 2)

Psychological distress was relatively high in PO and COSIP users at baseline, and after adjustment for the covariates, the LMM with an AR(1) covariance structure revealed a significant main effect of time for all three outcome measures (distress, depression and anxiety) (Tables 3 and 4). Distress scores showed a significant main effect of time, indicating that distress levels lowered across measurement occasions F(2, 107.24) = 14.72, *p* < .001. No significant main effect of group, F(1, 77.12) = 0.17, *p* = .678 or Time x Group F(2, 107.24) = 1.71, *p* = .186, was observed. Estimated marginal means (EMMs) for COSIP users were 7.21 (95% CI [6.45–7.96]) at baseline, 5.99 (95% CI [5.22–6.76]) after 6 months and 5.08 (95% CI [3.96–6.18]) after 12 months. PO users showed a similar change in distress with 7.29 (95% CI [6.54–8.05]) at baseline, 5.53 (95% CI [4.77–6.29]) after 6 months and 6 (95% CI [4.99–7.02]) after 12 months.

**Table 3:** Fixed effects from the linear mixed-effects model predicting psychological distress

	Effect	df1	df2	F	*p*
Distress	Time	2	107.24	14.72	< 0.001***
	Group	1	77.12	0.17	0.678
	Time x Group	2	107.24	1.71	0.186
Anxiety	Time	2	84.11	17.02	< 0.001***
	Group	1	73.10	0.18	0.672
	Time x Group	2	84.11	0.33	0.717
Depression	Time	2	69.61	12.27	< 0.001***
	Group	1	71.22	0	0.997
	Time x Group	2	69.61	2.62	0.80

**Table 4:** Estimated marginal means of scores by group and time point

		**Baseline**		**6 months**		**12 months**	
	Group	M (SE)	95% CI [lower - upper]	M (SE)	95% CI [lower - upper]	M (SE)	95% CI [lower - upper]
Distress	COSIP	7.2 (0.38)	[6.45–7.96]	5.99 (0.39)	[5.22–6.76]	5.08 (0.56)	[3.96–6.18]
	PO	7.29 (0.38)	[6.54–8.05]	5.53 (0.38)	[4.77–6.29]	6.00 (0.51)	[4.99–7.02]
Anxiety	COSIP	11.03 (0.84)	[9.36–12.70]	7.71 (0.84)	[6.04–9.37]	7.15 (1.14)	[4.90–9.39]
	PO	10.15 (0.84)	8.48–11.81]	7.77 (0.84)	[6.10–9.43]	6.65 (1.06)	[4.56–8.74]
Depression	COSIP	9.27 (0.91)	[7.46–11.07]	8.03 (0.91)	[6.22–9.84]	7.51 (1.19)	[5.16–9.85]
	PO	10.94 (0.91)	[9.14–12.75]	8 (0.91)	[6.19–9.81]	5.87 (1.11)	[3.68–8.07]

Anxiety scores also showed a significant main effect of time F(2, 84.11) = 17.02, *p* < .001 and no significant main effect of group, F(1, 73.10) = 0.18, *p* = .672 or Time x Group F(2, 84.11) = 0.33, *p* = .717. EMMs for COSIP were 11.03 (95% CI [9.36–12.70]) at baseline, 7.71 (95% CI [6.04–9.37]) after 6 month and 7.15 (95% CI [4.90–9.39]) after 12 months. PO users showed a similar reduction in anxiety with 10.15 (95% CI [8.48–11.81]) at baseline, 7.77 (95% CI [6.10–9.43]) after 6 months and 6.65 (95% CI [4.56–8.74]) after 12 months. Changes in depression scores indicate a decrease over time with a significant main effect F(2, 69.61) = 12.27, *p* < .001. Neither the main effect of group, F(1, 71.22) = 0.01, *p* = .997, nor the Time × Group interaction, F(2, 69.61) = 2.62, *p* = .80, reached statistical significance. EMMs indicated a decline in depression scores from baseline to follow-up in both groups. Although the PO group showed a numerically larger reduction (baseline: M = 10.94 95% CI [9.14–12.75], after 12 months: M = 5.87 95% CI [3.68–8.07]) than the COSIP group (baseline: M = 9.27, 95% CI [7.46–11.07]) after 12 months: M = 7.51, 95% CI [5.16–9.85]), this difference in change over time was not statistically significant. Fixed Effects from the LMM are reported in Table 3, EMMs are reported in Table 4.

### Family-related distress among COSIP users and its change over (Research question 3)

COSIP users reported significant changes over time for PCQ scores, parental HRQoL and the children’s HRQoL as shown in Table 5. No significant change over time was observed for FAD scores F(2, 28.51) = 0.05, *p* = .949. LMM revealed a significant effect of time on PCQ scores, F(2, 17.24) = 7.41, *p* = .006. EMMs showed a decrease in PCQ scores from baseline (M = 3.76, 95% CI [2.56, 4.95]) to the second assessment after 6 months (M = 3.20, 95% CI [2.01–4.38]) and the third assessment after 12 months (M = 2.90, 95% CI [1.67–4.12]). Parental HRQoL increased over time with 47.69 (95% CI [40.18–55.21]) at baseline, 56.16 (95% CI [48.44–63.87]) after 6 months and 62.70 (95% CI [52.01–73.32]) after 12 months, showing a significant effect of time F(2, 31.52) = 4.34, *p* = .022. In addition, children’s HRQoL changed significantly over time, F(2, 28.11) = 5.21, *p* = .012, with EMMs increasing from baseline (M = 42.60, 95% CI [36.51–48.69]) to 6 months (M = 50.76, 95% CI [44.99–56.52]) and 12 months (M = 56.31, 95% CI [49.30–63.32]) (Table 5).

**Table 5:** EMMs and LMM for changes of family-related distress among COSIP users over time

		EMMs		LMM changes over time	
	Time Point	M (SE)	95% CI [lower - upper]	F (df1, df2)	*p*
PCQ	Baseline	3.76 (0.59)	[2.56–4.95]	7.41 (2, 17.24)	0.006**
	6 months	3.20 (0.58)	[2.01–4.38]		
	12 months	2.90 (0.60)	[1.67–4.12]		
FAD	Baseline	1.86 (0.10)	[1.66–2.05]	0.05 (2, 28.51)	0.949
	6 months	1.84 (0.09)	[1.65–2.02]		
	12 months	1.96 (0.12)	[1.63–2.10]		
Parental HRQoL	Baseline	47.69 (3.74)	[40.18–55.21]	4.34 (2, 31.52)	0.022*
	6 months	56.16 (3.85)	[48.44–63.87]		
	12 months	62.70 (5.32)	[52.01–73.32]		
Children‘s HRQoL	Baseline	42.60 (3.02)	[36.51–48.69]	5.21 (2, 28.11)	0.012*
	6 months	50.76 (2.86)	[44.99–56.52]		
	12 months	56.31 (4.47)	[49.30–63.32]		

## Discussion

The present study examined psychological distress and family-related distress among families affected by parental cancer who use a preventive, child-centred family counselling based on longitudinal observational data. The study analysed data from 34 parents who had registered for COSIP, as well as from 34 matched parents who received PO.

Looking at psychological distress among COSIP and PO users (Research questions 1 and 2), baseline levels were elevated compared with available reference values, indicating a high burden in parents with cancer independent of the psycho-oncological support they use. These findings are consistent with prior research indicating that cancer patients who are parents report greater psychological burden than those without children, including higher levels of depressive symptoms and anxiety [10, 31, 32]. Compared to pre-pandemic findings [10, 28], parents in our sample reported even higher levels of psychological distress, comparable to reference values observed among parents with cancer during the COVID-19 pandemic [33]. The COVID-19 pandemic may have been an additional source of stress for these families. This could suggest that, during times of stressful life events such as the COVID-19 pandemic, support for parents with cancer is particularly important. Previous research indicates an association between parental stress and mental well-being in children [16]. Given that parental depression is a known risk factor for poorer outcomes in children [34, 35] and as we have observed elevated levels of depressive symptoms reported in both the COSIP and PO groups, family- and child-centred counselling could be relevant for all affected families in order to prevent negative long-term consequences for the children, given that studies provide evidence of positive effects [36, 37].

The adjusted models show a reduction in mean psychological distress scores over time in COSIP and PO users but no group effect. As we did not include a control group without any psychosocial support, the attribution to change over time to the interventions is not possible. Still, findings from systematic reviews suggest positive effects of psycho-oncological support for parents with cancer [37, 38]. The absence of statistically significant differences between groups may suggest that the type of psycho-oncological support—whether individual or family-centred—does not differentially affect parental stress levels.

Future studies should include a broader range of child-related outcomes to evaluate whether family-centred psycho-oncological interventions also benefit children, as evidence regarding child outcomes remains limited [37]. In addition, in our study, COSIP users were more often in partnerships and registered closer to the time of diagnosis for support than PO users. This pattern may plausibly reflect differences in needs and family structures. Parents in partnerships may be more likely to engage in dyadic and family-centred coping, which is more closely aligned with the COSIP approach. In addition, family-centred counselling may require greater organizational effort, which may be more difficult for single parents to accommodate. The earlier timing of support may indicate specific needs, for example regarding communication with children about the diagnosis. However, our data does not allow for further interpretation and longitudinal studies with parents along the cancer journey are needed.

Among COSIP users we observed an improvement in most family-related outcomes including HRQoL of parents and children over time (Research question 3). The HRQoL scores of parents reported by our sample are comparable to previously reported data for parents with cancer [10, 28] and lower than population-based norms [39, 40] The HRQoL scores reported for children in our study were numerically slightly lower than those reported in previous research [28, 41] and lower compared to the norm [42]. Whether this difference is related to the timing of our data collection during the COVID-19 pandemic or simply reflects random variation cannot be determined based on the present data. The present analysis showed that parents generally experienced at least some level of concern about the impact of the disease on their children. This aligns with previous research on parenting concerns from other samples of parents with cancer [43, 44] and may suggest that parenting concerns are common among parents with cancer and are not limited to those who actively seek family-centred counselling. These findings suggest that family-related stressors should be routinely assessed rather than identified only when parents explicitly express a need for counselling. Accordingly, family-centred support services may be relevant for a broader group of parents with cancer, including those who do not actively seek psycho-oncological counselling. Levels of general family functioning, as assessed with the FAD, remained stable over time in the present sample and fall within the range typically interpreted as “effective” family functioning. This pattern may reflect a selection bias. Families with at least moderately effective family functioning may be more likely to enroll in family counselling and complete follow-up assessments, whereas those with more severely impaired functioning may be underrepresented in the sample. Unfortunately, no data on family-related distress were available for the PO group. Findings from previous studies indicate that parents, independent from seeking for psycho-oncological support, experience family-related distress [28, 43]. Hence, future interventional studies may include family-related outcomes to identify these effects of psycho-oncological interventions although not explicitly being family- or child-centred.

### Study limitations

This study has several limitations that should be considered when interpreting the findings. Although COSIP and PO users were matched by age, gender, and role, relevant baseline differences remained regarding family status and time since initial diagnosis. As treatment allocation was not randomized, residual confounding due to unmeasured patient or clinical characteristics cannot be excluded. The naturalistic design may enhance external validity for the investigated clinical setting. Still, given the observational design and the absence of a control group without psycho-oncological care, we cannot determine whether the observed improvements over time are attributable to the support services, would have occurred naturally over the course of the illness or are influenced by other external factors that we did not assess. The relatively small sample size, the single centre design and the high attrition rate impeded analytic power, which limits the interpretation of findings and may further limit the generalisability beyond the investigated clinical setting. As usual for studies including routine care as comparator [45], information on the use of additional psychosocial support services outside the psycho-oncological outpatient clinic was unavailable, limiting the ability to account for potential confounding factors. Because family-related psychosocial variables were not assessed in the PO group, direct comparisons between groups for these outcomes were not possible. In addition, children’s outcomes were assessed using parental proxy reports, which may be subject to reporting bias and may not fully capture children’s own perspectives. Future multi-centre studies with larger and more representative samples, including an appropriate comparison group and comprehensive assessment of relevant psychosocial factors and family-related outcomes, are needed to confirm and extend our findings.

### Clinical implications

The findings indicate that parents in families affected by parental cancer raising minor children that register for individual or family-centred psycho-oncological support during the COVID-19 pandemic experienced considerable psychological distress. Distress levels decline over the time of 12 months after initial registration for support and our findings suggest no differences between COSIP and PO users regarding the trajectory of psychological distress in our sample. To best allocate patients according to their primary psycho-social needs profile, family-related distress should be assessed along with psychological distress. Given the high levels of psychological symptoms at the first measurement point in our sample when registering, and given that children of parents with cancer reporting high psychological burden are at particular risk for developing psychosocial problems of their own, a family-centred approach could be considered for those patients.

### Conclusion

In conclusion, cancer patients with minor children who experience additional stressors such as the COVID-19 pandemic and who actively seek psycho-oncological or family-focused support may represent a particularly burdened subgroup. The present findings suggest that this group initially reports high levels of psychological distress and that mean levels of distress, anxiety, and depressive symptoms decrease over time. Against the background of current guidelines of psychosocial support in cancer patients, the findings highlight the relevance of psycho-oncological care and family-centred counselling to address the psychological distress and specific family-related needs. Within the limitations of this observational study, the findings suggest that offering different psychosocial care pathways may be relevant for parents affected by cancer, without providing evidence that one format is superior to the other.

Given the methodological limitations of our study, further research with larger, preferably multicentre samples is required to more precisely examine potential intervention effects and further investigate potential differences between individual and family-centred support (e.g. in family-related outcomes).

## Supplementary Information

Supplementary Material 1: Appendix A.

Supplementary Material 2: STROBE checklist.

## Data Availability

The datasets used and/or analysed during the current study are available from the corresponding author on reasonable request.

## Abbreviations

COSIP: Children of somatically ill Parents
EMMs: Estimated marginal means
EORTC QLQ: European Organization for Research and Treatment of Cancer Quality of Life
FAD: Family Assessment Device
GAD: Generalized Anxiety Disorder
GF: General Functioning subscale
HRQoL: Health Related Quality of Life
LMM: Linear mixed-effects model
NCCN: National Comprehensive Cancer Network
PCQ: Parenting Concerns Questionnaire
PHQ: Patient Health Questionnaire
PO: Psycho-oncological Care
STROBE: STrengthening the Reporting of OBservational studies in Epidemiology
QoL: Quality of Life
