# Supplementary material for: Family-centred psycho-oncological counselling for parents with cancer: a longitudinal secondary data analysis of psychological distress, family functioning and parental concerns
Source: BMC Cancer. 2026 Sep 22;26:1131. doi: 10.1186/s12885-026-17004-z (PMC13595782; doi:10.1186/s12885-026-17004-z)
Supplement: Supplementary file 1 [file 12885_2026_17004_MOESM1_ESM.docx]

| Appendix A  Table 1 drop-out analysis | |  |  |  |  |  |  |  |
| --- | --- | --- | --- | --- | --- | --- | --- | --- |
|  |  |  |  |  |  |  |  |  |
| 6-month follow-up |  |  |  |  |  |  |  |  |
|  | Questionnaire completed and returned (t0 and t1) | | | No Questionnaire returned at 6-month follow-up (t1) | | | t-test | |
| Variable | N | mean | SD | N | mean | SD | p | cohen's d |
| Age | 34 | 44.59 | 6.28 | 70 | 45.38 | 7.67 | .571 | .108 |
| Number of children | 34 | 1.94 | 0.65 | 70 | 1.9 | 0.85 | .761 | .057 |
| Distress (DT) | 34 | 7.21 | 2.21 | 70 | 6.99 | 1.95 | .622 | .108 |
| Anxiety (GAD-7) | 34 | 10.94 | 5.96 | 70 | 9.84 | 5.29 | .359 | .199 |
| Depressive Symptoms (PHQ-9) | 34 | 9.27 | 5.17 | 70 | 9.26 | 5.25 | .996 | .001 |
| Parental concerns (PCQ) | 20 | 2.99 | 0.61 | 84 | 3.24 | 0.77 | .169 | .329 |
| Family funtioning (FAD) | 20 | 1.90 | 0.70 | 84 | 2.04 | 0,64 | .459 | .205 |
| Global QoL (EORTC-30) | 34 | 44.85 | 24.19 | 70 | 50.87 | 17.91 | .199 | .300 |
|  |  |  |  |  |  |  |  |  |
| 12-month follow-up |  |  |  |  |  |  |  |  |
|  | Questionnaire completed and returned (t0, t1, t2) | | | No Questionnaire returned at 12-month follow-up (t2) | | | t-test | |
| Variable | N | mean | SD | N | mean | SD | p | cohen's d |
| Age | 15 | 44.20 | 7.54 | 19 | 44.89 | 5.27 | .764 | .109 |
| Number of children | 15 | 1.87 | 0.52 | 19 | 2 | 0.75 | .560 | .204 |
| Distress (DT) | 15 | 7.4 | 2.03 | 19 | 7.05 | 2.39 | .656 | .155 |
| Anxiety (GAD-7) | 15 | 10.47 | 6.22 | 19 | 11.32 | 5.89 | .687 | .141 |
| Depressive Symptoms (PHQ-9) | 15 | 8.67 | 3.52 | 19 | 9.74 | 6.23 | .557 | .205 |
| Parental concerns (PCQ) | 8 | 3.01 | 0.73 | 12 | 2.96 | 0.55 | .745 | .547 |
| Family funtioning (FAD) | 8 | 2.23 | 0.89 | 12 | 1.69 | 0.47 | .246 | .151 |
| Global QoL (EORTC-30) | 15 | 49.44 | 19.54 | 19 | 48.95 | 22.12 | .949 | .023 |

| Table 2 |  |  |  |  |
| --- | --- | --- | --- | --- |
| Pairwise comparison family-related distress among COSIP users over time | | | | |
|  | Comparison | Mean difference | 95 % CI | p (Bonferroni) |
| PCQ | 6 months - Baseline | -0.56 | [-0.99 - -0.13] | **.009**** |
|  | 12 months - Baseline | -0.86 | [-1.50 - -0.22] | **.010*** |
| FAD | 6 months - Baseline | -0.21 | [-0.22 - 0.18] | 1 |
|  | 12 months - Baseline | 0.01 | [-0.29 - 0.30] | 1 |
| Parent HRQoL | 6 months - Baseline | 8.47 | [0.09 - 16.85] | **.047*** |
|  | 12 months - Baseline | 15.01 | [1.47 - 28.54] | **.028*** |
| Child HRQoL | 6 months - Baseline | 8.16 | [-1.25 - 17.57] | .099 |
|  | 12 months - Baseline | 13.71 | [3.20 - 24.22] | **.010*** |
