# Supplementary material for: Family-centred psycho-oncological counselling for parents with cancer: a longitudinal secondary data analysis of psychological distress, family functioning and parental concerns
Source: BMC Cancer. 2026 Sep 22;26:1131. doi: 10.1186/s12885-026-17004-z (PMC13595782; doi:10.1186/s12885-026-17004-z)
Supplement: Supplementary file 2 [file 12885_2026_17004_MOESM2_ESM.docx]

STROBE Statement—checklist of items that should be included in reports of observational studies

|  | Item No. | Recommendation | Page  No. | Relevant text from manuscript |
| --- | --- | --- | --- | --- |
| **Title and abstract** | 1 | (*a*) Indicate the study’s design with a commonly used term in the title or the abstract | 1 | Family-centred psycho-oncological counselling for parents with cancer: A longitudinal secondary data analysis of psychological distress, family functioning and parental concerns. |
|  |  | (*b*) Provide in the abstract an informative and balanced summary of what was done and what was found | 2 | Methods  We conducted a secondary, longitudinal observational study based on routine care data from users of an outpatient psycho‐oncological clinic. The database comprised assessments at baseline and at 6- and 12-month follow-ups, including measures of distress, anxiety and depression for COSIP and PO users. For COSIP users additional data on health-related quality of life (HRQoL), parental concerns, and family functioning was included. We conducted descriptive analyses and examined baseline group differences using t-tests and Pearson’s chi-square tests. Longitudinal trajectories were analysed using linear mixed-effects models.  Results  Data from n = 34 parents who had registered themselves and their families for a COSIP counselling session between 2020 and 2022 were included in the analyses. Among the COSIP users, the time since the initial diagnosis was shorter on average (p = .017) and the parents were separated less frequently (p = .015) than in the group of PO users (n = 26). At baseline, PO and COSIP users exhibited elevated levels of psychological burden, with over 80% of parents reporting clinically significant distress, 54% presenting with moderate to severe anxiety symptoms, and 50% showing elevated levels of depressive symptoms. In the adjusted model, a significant main effect of time, for distress F(2, 107.24) = 14.72, p < .001, depression F(2, 69.61) = 12.27, p < .001 and anxiety F(2, 84.11) = 17.02, p < .001, but no group difference were found. We identified a significant effect of time for parental concerns F(2, 17.24) = 7.41, p = .006, parental HRQoL F(2, 31.52) 0 4.34, p = .022 and children’s HRQoL F(2, 28.11) = 5.21, p = .012 among COSIP users. |
| Introduction | | | |  |
| Background/rationale | 2 | Explain the scientific background and rationale for the investigation being reported | 4-5 | About one in five people develop cancer in their lifetime^1^. Prevalence rates indicate that between 14 and 25% of cancer patients are parenting minor children. Thus, estimates reveal between 1.6 and 8.4% of children and adolescents have a parent with a history of cancer^2^. Families affected by cancer face a variety of new situations and challenges. The illness places a burden on all family members. However, children’s adaptation depends less on the cancer itself and more on the family’s coping processes and resources^3^. In families, uncertainties arise, for example, in the context of parent-child communication, especially concerning the illness^4,5^ and in communicating and dealing with individual needs^6^. Parents often notice signs of distress in their children, but sometimes avoid talking about their emotional reactions^7^. Previous research suggested that a cohesive, communicative and collaborative family system could have a protective effect on children’s mental well-being^8^. In addition, parents with cancer experience substantial parenting distress, including concerns about their children's well-being, maintaining family roles, and coping with illness-related demands^9^. A systematic review also revealed significant psychological distress, with high rates of anxiety and depressive symptoms among cancer patients parenting minor children^10^.  In order to meet the situation of affected families and to support them, specific counselling and care services exist nationally and internationally^11-14^. One of those interventions is the COSIP-concept (Children of somatically ill parents). It was developed in 2007 as a manualized, preventive, supportive intervention for families with a parent who is physically ill and was further specialized for the psycho-oncological setting in 2014^3^. The programme aims to support children, parents, and the family system as a whole by addressing illness-related communication, parental concerns, family functioning, and children’s psychosocial needs. Further information on the COSIP concept can be found in Thastum et al.^15^ and Romer et al.^3^. A setting for the whole family is particularly important as good communication and a supportive family environment that understands everyone's needs appear to be important protective factors that facilitate the entire family's adjustment to the illness^16^.  Although family-focused interventions for families affected by parental cancer have been implemented for some time and are generally perceived as helpful, acceptable and feasible, a recent review indicates that only few interventions have undergone comprehensive evaluation, and evidence regarding the long-term sustainability of improvements in outcomes remains limited^9^. |
| Objectives | 3 | State specific objectives, including any prespecified hypotheses | 6 | The following research questions were addressed:   I. What is the level of psychological distress (including distress, depressive symptoms, and anxiety) among parents with minor children and a parental cancer diagnosis who register for COSIP? II. How does psychological distress among COSIP users change over time, and how does it compare with users receiving routine psycho-oncological care (PO users)? III. What are the levels of family-specific distress (including parental concerns, family functioning, parental health-related quality of life (HRQoL) and children’s HRQoL) among COSIP users, and how does the family-specific distress change over time? |
| Methods | | | |  |
| Study design | 4 | Present key elements of study design early in the paper | 6 | An exploratory secondary analysis was performed using longitudinal observational data derived from routine assessments conducted at baseline and at 6- and 12-month follow-up. The study employed a naturalistic design and included a sample of parents who enrolled in COSIP, alongside a matched comparison group of PO users at the specialized outpatient clinic. COSIP users were matched with PO users through a matching process based on age, gender, and role (patient versus healthy partner). |
| Setting | 5 | Describe the setting, locations, and relevant dates, including periods of recruitment, exposure, follow-up, and data collection | 3, 9-11 | **Interventions**  COSIP and PO are part of the specialist care provided in an outpatient clinic at a comprehensive cancer centre in Germany.  ***COSIP – child-centred counselling for families***  The conceptual framework for child-centred counselling for families affected by parental cancer was developed on the basis of the research conducted by the COSIP group^15^. The COSIP approach provides a flexible intervention framework that is tailored to each family’s individual needs, allowing both the number of sessions and the content to vary accordingly. The intervention is characterised by three phases: a diagnostic phase and an interventional phase, which comprises approx. 3–8 sessions, and transitions into a flexible concluding phase^3^. The manual provides specific guidance on conducting initial diagnostic interviews and formulating clearly defined intervention objectives. These objectives are tailored to the specific needs of each family. The focus of counselling can be on the family as a whole (e.g., promoting open communication), on the parents (e.g., strengthening perceived parenting skills) or on the child (e.g., strengthening the child's active coping resources). COSIP counselling is provided by a child and adolescent psychotherapist or a psychotherapist who has completed training in the COSIP concept.  ***PO - Individual psycho‐oncological treatment of the comparison group***  The psycho‐oncological outpatient clinic offers supportive psychotherapeutic care to cancer patients and their relatives, primarily delivered by clinical psychologists and certified psychotherapists trained in behavioural, humanistic therapy, or psychodynamic psychotherapy. Treatment mainly consists of one-on-one sessions with cancer patients or relatives, or may involve group sessions, couple sessions, or art and music therapy. Information on the specific treatments received by the patients included in this analysis was not available within the dataset. |
| Participants | 6 | (a) Cohort study—Give the eligibility criteria, and the sources and methods of selection of participants. Describe methods of follow-up  Case-control study—Give the eligibility criteria, and the sources and methods of case ascertainment and control selection. Give the rationale for the choice of cases and controls  Cross-sectional study—Give the eligibility criteria, and the sources and methods of selection of participants | 9 | The participants comprised a sample of parents who were responsible for minor children and who had attended either at least one COSIP counselling session between January 2020 and December 2022 or at least one general PO appointment, and for whom were available both at the time of the baseline survey and at 6-months follow-up. No restrictions were applied regarding cancer type, disease stage, family composition, or the nature of counselling concerns. As the questionnaire was part of a routine assessment and solely available in German, sufficient language skills were required for completion. |
|  |  | (b) Cohort study—For matched studies, give matching criteria and number of exposed and unexposed  Case-control study—For matched studies, give matching criteria and the number of controls per case | 10 | Parents with minor children receiving PO were selected as a comparison group and descriptively matched to COSIP users based on age, gender, and role (patient or healthy partner). During the matching process, each parent in the COSIP group was matched with a parent from the PO group who corresponded in terms of these characteristics. Where possible, parents were matched directly by age; if an exact age match was not available, parents were matched within predefined 5-year age categories (e.g., 40–44 years). |
| Variables | 7 | Clearly define all outcomes, exposures, predictors, potential confounders, and effect modifiers. Give diagnostic criteria, if applicable | 7-9 | Measures  Demographic and disease‐related variables  Demographic and disease-related data were obtained via self-report. Demographic variables comprised age, sex, number and age of children, and marital status. Disease-related variables included cancer diagnosis, presence of metastases, and time since diagnosis.  Psychological distress (assessed among COSIP and PO users)  Distress  The NCCN Distress thermometer is a screening instrument developed by the National Comprehensive Cancer Network (NCCN) to assess psychosocial distress in oncological patients. It consists of a scale from 0 to 10, on which the experienced distress of the last week is reported. The internationally recommended cut-off of 5 or more was used as an indicator that a patient is in distress and needs support.  Depressive Symptoms  Depressive symptoms were assessed using the PHQ‐9. All 9 items can be rated on a 4‐point Likert‐scale from 0 to 3. The PHQ‐9 has good reliability and validity with higher sum scores indicating higher severity of depressive symptoms (total score range 0‐27). A total score of 10 or higher indicates possible depressive disorder.  Anxiety  Anxiety was assessed with the GAD‐7, which uses a 4‐point Likert‐scale from 0 to 3. The GAD‐7 shows good psychometric properties. Total score range is 0-21 and higher sum scores indicate higher severity of anxiety symptoms. A total score of 10 to 14 indicates moderate anxiety symptoms, and a total score of 15 and higher indicates severe symptom levels.  Family-related distress (only assessed among COSIP users)  HRQoL  The HRQoL among COSIP users was measured using the German version of the global scale of the European Organization for Research and Treatment of Cancer Quality of Life Questionnaire (EORTC QLQ-C30). We used the Global Quality of Life scale as outcome, which is calculated by adding up the responses to two specific questions (question 29 and question 30) and transforming this sum linearly into a range from 0 to 100 with higher scores indicating better HRQoL. The EORTC QLQ-C30 is known to be reliable and valid and is widely used.  Family Functioning  Family functioning indicates the extent to which the family responds to the needs of its members. The German version of the General Functioning subscale (GF12) of The McMaster Family Assessment Device (FAD) was used to assess family functioning. It consists of 12 items and it can be used alone as a global index and a valid general family measure. It is scored on a Likert scale from 1 to 4, higher scores represent more problems. The FAD shows good acceptance, reliability and validity.  Parenting concerns  The parenting concerns of cancer patients were measured with the German version of the Parenting Concerns Questionnaire (PCQ). The scale consists of 15 items that can be rated on a scale from 1 (not concerned) to 5 (extremely concerned). The PCQ proved a reliable and valid measure of parenting distress among cancer patients.  Children’s HRQoL  We assessed the children’s HRQoL using the proxy version of the KIDSCREEN-10 Index. Parents answered the 10 items on a 5-point Likert scale (1 = not at all to 5 = extremely). We transformed the scores into T-values (M = 50, SD = 10, possible range 0-100) according to the scoring manual. Higher scores represent a higher health-related quality of life. Research has shown that the KIDSCREEN-10 is a reliable, valid, sensitive, and conceptually appropriate Quality of Life measure. Although the original version of the KIDSCREEN-10 is intended for school children aged 6 to 18 years, we have also used it for younger children as other researchers have successfully done before. |
| Data sources/ measurement | 8* | For each variable of interest, give sources of data and details of methods of assessment (measurement). Describe comparability of assessment methods if there is more than one group | 6-9 | See description above |
| Bias | 9 | Describe any efforts to address potential sources of bias | 10 | Despite the matching procedure, COSIP and PO users differed significantly at baseline regarding family status (p = .015, Cramer’s V = 0.35) and time since initial diagnosis (p = .017, d = 0.60) (Table 1). To account for these residual baseline differences, family status and time since diagnosis were included as covariates in the linear mixed-effects model (LMM). Therefore, between-group effects were estimated while statistically adjusting for these baseline characteristics. |
| Study size | 10 | Explain how the study size was arrived at | 10 | The participants comprised a sample of parents who were responsible for minor children and who had attended either at least one COSIP counselling session between January 2020 and December 2022 or at least one general PO appointment, and for whom were available both at the time of the baseline survey and at 6-months follow-up. |

| Quantitative variables | 11 | Explain how quantitative variables were handled in the analyses. If applicable, describe which groupings were chosen and why | 11 | Descriptive analyses were performed to characterise the study sample and to identify the level of psychological distress (including distress, depressive symptoms, and anxiety) of COSIP users at time of registration (Research question 1) and follow-up assessments. To compare COSIP and PO users (Research question 2) trajectories of psychological distress (distress, anxiety, depression) across the three measurement time points were examined using LMM. The models included group, time, and the group-by-time interaction as fixed effects. Family status and time since first diagnosis were included as covariates to consider baseline differences between groups. Participant was modelled as a random intercept to account for the dependency of repeated observations within individuals. LMM were estimated using restricted maximum likelihood (REML). Repeated measurements were modelled with a first-order autoregressive [AR(1)] covariance structure. Family-related distress among COSIP users over time was analysed using LMM with a random intercept for participant to account for within-person correlations across three time points (Research question 3). The significance level was set at α = .05. Statistical analyses were performed using IBM SPSS Statistics Version 29.0.  s |
| --- | --- | --- | --- | --- |
| Statistical methods | 12 | (*a*) Describe all statistical methods, including those used to control for confounding | 12 |  |
|  |  | (*b*) Describe any methods used to examine subgroups and interactions | 12 |  |
|  |  | (*c*) Explain how missing data were addressed | 11 | Missing data handling followed the scoring recommendations provided in the respective manuals for the EORTC QLQ-C30 and KIDSCREEN-10. For the PHQ-9, missing items were handled in accordance with previous research using the instrument, where missing values were replaced by the mean of the remaining items if fewer than 20% of items were missing29. This procedure was also applied to the PCQ, GAD-7 and FAD scales, given the low proportion of missing values30. The rationale for this approach was to maximize the available sample size while minimizing potential bias introduced by exclusion of participants through listwise deletion. |
|  |  | (*d*) *Cohort study*—If applicable, explain how loss to follow-up was addressed  *Case-control study*—If applicable, explain how matching of cases and controls was addressed  *Cross-sectional study*—If applicable, describe analytical methods taking account of sampling strategy | 11 | Matching COSIP and PO group  Parents with minor children receiving PO were selected as a comparison group and descriptively matched to COSIP users based on age, gender, and role (patient or healthy partner). During the matching process, each parent in the COSIP group was matched with a parent from the PO group who corresponded in terms of these characteristics. Where possible, parents were matched directly by age; if an exact age match was not available, parents were matched within predefined 5-year age categories (e.g., 40–44 years). |
|  |  | (*e*) Describe any sensitivity analyses |  |  |
| Results | | | | |
| Participants | 13* | (a) Report numbers of individuals at each stage of study—eg numbers potentially eligible, examined for eligibility, confirmed eligible, included in the study, completing follow-up, and analysed | 12 | A total of 176 families registered for COSIP counselling between January 2020 and December 2022. 38 cases had to be excluded from the analyses due to the parent's death prior to counselling, the parent having another life-threatening disease not cancer, or because instead of a parent, a sibling was diagnosed with cancer. 34 participants failed to respond after registration, while a further group did not complete the baseline questionnaire. Of the remaining 104 patients, 34 completed at least the first follow-up assessment and were consequently included in the analysis (Figure1). |
|  |  | (b) Give reasons for non-participation at each stage |  |  |
|  |  | (c) Consider use of a flow diagram |  | Figure 1 |
| Descriptive data | 14* | (a) Give characteristics of study participants (eg demographic, clinical, social) and information on exposures and potential confounders | Table | Table 1  In the majority of included COSIP users (79%, n = 27), the ill parent registered for COSIP themselves. The participants were mostly female (82%, n = 28) and well educated, with over 60% of them having a General Qualification for university entrance (n = 21). The mean age of the parent who completed the questionnaire was 45 years (SD = 6.3, range 34-56). The mean number of children per family was 2 (range 1-4). The initial diagnosis occurred mostly within the last year (71%, n = 24) or the past five years (26%, n = 8). Breast cancer with 44% (n = 15), lymphoma with 12% (n = 4) and brain cancer with 9 % (n = 3) were the most frequent cancer types. The mean number of counselling sessions was 5 (SD = 4.9, range 1-18) (Table 1). |
|  |  | (b) Indicate number of participants with missing data for each variable of interest |  |  |
|  |  | (c) *Cohort study*—Summarise follow-up time (eg, average and total amount) |  | Patients who wanted to attend COSIP counselling or PO were requested to complete and return a baseline questionnaire prior to their initial consultation. 6 months and 12 months following the initiation of counselling. |
| Outcome data | 15* | *Cohort study*—Report numbers of outcome events or summary measures over time |  |  |
|  |  | *Case-control study—*Report numbers in each exposure category, or summary measures of exposure |  |  |
|  |  | *Cross-sectional study—*Report numbers of outcome events or summary measures |  |  |
| Main results | 16 | (*a*) Give unadjusted estimates and, if applicable, confounder-adjusted estimates and their precision (eg, 95% confidence interval). Make clear which confounders were adjusted for and why they were included |  | s. Table 4 and Table 5  Psychological distress over time among COSIP users compared with PO users (Research question 2)  Psychological distress was relatively high in PO and COSIP at baseline, and a LMM with an AR(1) covariance structure revealed a significant main effect of time for all three outcome measures (distress, depression and anxiety) (Table 3 and Table 4). Distress scores showed a significant main effect of time, indicating that distress levels lowered across measurement occasions F(2, 2, 107.24) = 14.72, p < .001. No significant main effect of group, F(1, 77.12) = 0.17, p = .678 or Time x Group F(2, 107.24) = 1.71, p = .186, was observed. Estimated marginal means (EMMs)for COSIP were 7.21 (95% CI [6.45 - 7.96]) at baseline, 5.99 (95% CI [5.22 - 6.76]) after 6 month and 5.08 (95% CI [3.96 - 6.18]) after 12 months. PO users showed a similar change in distress with 7.29 (95% CI [6.54 - 8.05]) at baseline, 5.53 (95% CI [4.77 - 6.29]) after 6 months and 6 (95% CI [4.99 - 7.02]) after 12 months.  Anxiety scores also showed a significant main effect of time F(2, 84.11) = 17.02, p < .001 and no significant main effect of group, F(1, 73.10) = 0.18, p = .672 or Time x Group F(2, 84.11) = 0.33, p = .717. EMMs for COSIP were 11.03 (95% CI [9.36 - 12.70]) at baseline, 7.71 (95% CI [6.04 - 9.37]) after 6 month and 7.15 (95% CI [4.90 - 9.39]) after 12 months. PO users showed a similar reduction in anxiety with 10.15 (95% CI [8.48 - 11.81]) at baseline, 7.77 (95% CI [6.10 - 9.43]) after 6 months and 6.65 (95% CI [4.56 - 8.74]) after 12 months. Changes in depression scores indicate a decrease over time with a significant main effect F(2, 69.61) = 12.27, p < .001. Neither the main effect of group, F(1, 71.22) = 0.01, p = .997, nor the Time × Group interaction, F(2, 69.61) = 2.62, p = .080, reached statistical significance. EMMs indicated a decline in depression scores from baseline to follow-up in both groups. Although the PO group showed a numerically larger reduction (baseline: M = 10.94 95% CI [9.14 - 12.75], after 12 months: M = 5.87 95% CI [3.68 - 8.07]) than the COSIP group (baseline: M = 9.27, 95% CI [7.46 - 11.07]) after 12 months: M = 7.51, 95% CI [5.16 - 9.85]), this difference in change over time was not statistically significant. Fixed Effects from the LMM are reported in Table 3, EMMs are reported in Table 4.  Family-related distress among COSIP users and its change over (Research question 3)  COSIP users reported significant changes over time for PCQ scores, parental HRQoL and the proxy score of the children’s HRQoL as shown in Table 5. No significant change over time was observed for FAD scores F(2, 28.51) = 0.05, p = .949. LMM revealed a significant effect of time on PCQ scores, F(2, 17.24) = 7.41, p = .006. EMMs showed a decrease in PCQ scores from baseline (M = 3.76, 95% CI [2.56, 4.95]) to the second assessment after 6 months (M = 3.20, 95% CI [2.01 - 4.38]) and the third assessment after 12 months (M = 2.90, 95% CI [1.67 - 4.12]). Parental HRQoL increased over time with 47.69 (95% CI [40.18 - 55.21]) at baseline, 56.16 (95% CI [48.44 - 63.87]) after 6 months and 62.70 (95% CI [52.01 - 73.32]) after 12 months, showing a significant effect of time F(2, 31.52) = 4.34, p = 0.22. In addition, children’s HRQoL changed significantly over time, F(2, 28.11) = 5.21, p = .012, with EMMs increasing from baseline (M = 42.60, 95% CI [36.51 - 48.69]) to 6 months (M = 50.76, 95% CI [44.99 - 56.52]) and 12 months (M = 56.13, 95% CI [49.30 - 63.32]) (Table 5). |
|  |  | (*b*) Report category boundaries when continuous variables were categorized | Table 2 | The level of general psychological stress among COSIP users was high at baseline and decreased over time. Based on established cut-off values, 82% of COSIP users met the criterion for elevated distress at baseline, with this proportion decreasing to 65% at 6 months follow up and 24% at 12 months follow up. Moderate to severe anxiety symptoms were reported by 50% at baseline, 32% at 6 months follow up, and 12% at 12 months follow up; corresponding values for depressive symptoms were 44%, 32%, and 9%, respectively (Table 2). |
|  |  | (*c*) If relevant, consider translating estimates of relative risk into absolute risk for a meaningful time period |  |  |

| Other analyses | 17 | Report other analyses done—eg analyses of subgroups and interactions, and sensitivity analyses |  |  |
| --- | --- | --- | --- | --- |
| Discussion | | | | |
| Key results | 18 | Summarise key results with reference to study objectives | 15 | **Descriptive analysis of psychological distress among COSIP users (Research question 1)**  The level of general psychological stress among COSIP users was high at baseline and decreased over time.  **Psychological distress over time among COSIP users compared with PO users** (Research question 2)  Psychological distress was relatively high in PO and COSIP users at baseline, and after adjustment for the covariates, the LMM with an AR(1) covariance structure revealed a significant main effect of time for all three outcome measures (distress, depression and anxiety) (Table 3 and Table 4).  **Family-related distress among COSIP users and its change over (Research question 3)**  COSIP users reported significant changes over time for PCQ scores, parental HRQoL and the children’s HRQoL as shown in Table 5 |
| Limitations | 19 | Discuss limitations of the study, taking into account sources of potential bias or imprecision. Discuss both direction and magnitude of any potential bias | 18 | This study has several limitations that should be considered when interpreting the findings. Although COSIP and PO users were matched by age, gender, and role, relevant baseline differences remained regarding family status and time since initial diagnosis. As treatment allocation was not randomized, residual confounding due to unmeasured patient or clinical characteristics cannot be excluded. The naturalistic design may enhance external validity for the investigated clinical setting. Still, given the observational design and the absence of a control group without psycho-oncological, we cannot determine whether the observed improvements over time are attributable to the support services, would have occurred naturally over the course of the illness or are influenced by other external factors that we did not assess. The relatively small sample size, the single centre design and the high attrition rate impeded analytic power, which limits the interpretation of findings and may further limit the generalisability beyond the investigated clinical setting. As usual for studies including routine care as comparator45, information on the use of additional psychosocial support services outside the psycho-oncological outpatient clinic was unavailable, limiting the ability to account for potential confounding factors. Because family-related psychosocial variables were not assessed in the PO group, direct comparisons between groups for these outcomes were not possible. In addition, children’s outcomes were assessed using parental proxy reports, which may be subject to reporting bias and may not fully capture children’s own perspectives. Future multi-centre studies with larger and more representative samples, including an appropriate comparison groups and comprehensive assessment of relevant psychosocial factors and family-related outcomes, are needed to confirm and extend our findings. |
| Interpretation | 20 | Give a cautious overall interpretation of results considering objectives, limitations, multiplicity of analyses, results from similar studies, and other relevant evidence | 19 | In conclusion, cancer patients with minor children who experience additional stressors such as the COVID-19 pandemic and who actively seek psycho-oncological or family-focused support may represent a particularly burdened subgroup. The present findings suggest that this group initially reports high levels of psychological distress and that mean levels of distress, anxiety, and depressive symptoms decrease over time Against the background of current guidelines of psychosocial support in cancer patients, the findings highlight the relevance of psycho-oncological care and family-centred counselling to address the psychological distress and specific family-related needs. Within the limitations of this observational study, the findings suggest that offering different psychosocial care pathways may be relevant for parents affected by cancer, without providing evidence that one format is superior to the other.  Given the methodological limitations of our study, further research with larger, preferably multicentre samples is required to more precisely examine potential intervention effects and further investigate potential differences between individual and family-centred support (e.g. in family-related outcomes). |
| Generalisability | 21 | Discuss the generalisability (external validity) of the study results | 18 | The single-centre design may further limit generalisability beyond the investigated clinical setting |
| Other information | |  | | |
| Funding | 22 | Give the source of funding and the role of the funders for the present study and, if applicable, for the original study on which the present article is based | 22 | This research did not receive any specific grant from funding agencies in the public, commercial, or not-for-profit sectors. |

*Give information separately for cases and controls in case-control studies and, if applicable, for exposed and unexposed groups in cohort and cross-sectional studies.

**Note:** An Explanation and Elaboration article discusses each checklist item and gives methodological background and published examples of transparent reporting. The STROBE checklist is best used in conjunction with this article (freely available on the Web sites of PLoS Medicine at http://www.plosmedicine.org/, Annals of Internal Medicine at http://www.annals.org/, and Epidemiology at http://www.epidem.com/). Information on the STROBE Initiative is available at www.strobe-statement.org.
